# Sequence to structural analysis of ORF5 protein in Norway rat Hepatitis E Virus

**DOI:** 10.6026/97320630018019

**Published:** 2022-01-31

**Authors:** Zoya Shafat, Anwar Ahmed, Mohammad K Parvez, Shama Parveen

**Affiliations:** 1 Centre for Interdisciplinary Research in Basic Sciences, Jamia Millia Islamia, New Delhi, India; 2 Centre of Excellence in Biotechnology Research, College of Science, King Saud University, Riyadh, Saudi Arabia; 3 Department of Pharmacognosy, College of Pharmacy, King Saud University, Riyadh, Saudi Arabia

**Keywords:** Rat HEV, open reading frame 5 (ORF5), physicochemical parameters, primary structure, secondary structure, homology modelling, tertiary structure, motif prediction

## Abstract

Hepatitis E virus (HEV) is a major causative agent of acute hepatitis in developing countries. The Norway rat HEV genome consists of six open reading frames (ORFs), i.e., ORF1, ORF2, ORF3, ORF4, ORF5 and ORF6. The additional reading frame encoded protein ORF5 is attributed to life cycle of rat HEV. The ORFF5 protein’s function remains undetermined. Therefore, it is of interest to analyze the ORF5 protein for its physiochemical properties, primary structure, secondary structure, tertiary structure and functional characteristics using bioinformatics tools. Analysis of the ORF5 protein revealed it as highly unstable, hydrophilic with basic pI. The ORF5 protein consisted mostly of Arg, Pro, Ser, Leu and Gly. The 3D structural homology model of the ORF5 protein generated showed mixed α/β structural fold with predominance of coils. Structural analysis revealed the presence of clefts, pores and a tunnel. This data will help in the sequence, structure and functional annotation of ORF5.

## Background:

Hepatitis E virus (HEV) is the major aetiological agent of Hepatitis E, also called enteric hepatitis (enteric means related to the intestines) infection [1]. Worldwide, about 20 million HEV infections and 3.3 million symptomatic hepatitis E cases occur annually which results in 44,000 deaths [2]. HEV belongs to the family Hepeviridae and genus Orthohepevirus [3]. The HEV genome is a single, positive-sense RNA (7.2 kb in length), which is flanked with short 5' and 3' non-coding regions (NCR) [4]. The HEV genome is categorized into three open reading frames (ORFs): ORF1, ORF2 and ORF3. The ORF1, ORF2 and ORF3 encode the non-structural polyprotein (pORF1), capsid protein (pORF2) and the pleotropic protein (pORF3) respectively [5].

Hepeviruses belonging to the Hepeviridae family is classified into two genera: Orthohepevirus and Piscihepevirus [6,7]. Genus Orthohepevirus consists of four species: Orthohepevirus A–D. Within the Orthohepevirus A species, till date 8 separate genotypes (GT) (HEV-1 to HEV-8) and numerous sub-genotypes have been recognized [6,7]. Recent studies have reported that members of the Orthohepevirus C species (HEV-C1) are also pathogenic to humans. Interestingly, genetically highly divergent rodent-associated hepevirus was discovered from fecal and liver specimens from Norway rats of Germany in the year 2009 [8] and has been classified into species Orthohepevirus C genotype HEV-C1. In the year 2009, two complete nucleotide sequences were analyzed from Norway rats in Germany which suggested a completely separate genotype for these HEV strains [8]. These nucleotide sequences had high divergence to other HEV strains, i.e., HEV G1, HEV G2, HEV G3, HEV G4 and avian HEV [8]. It was predicted through software that the genome in these rat HEV sequences was organized into a total of six reading frames (ORF1, ORF2, ORF3, ORF4, ORF5 and ORF6). i.e., rat HEV genome consisted of three additional ORFs (ORF4, ORF5 and ORF6). It was also identified that unlike typical HEV genomic organization, the ORFs ORF1 and ORF3 do not overlap in these two rat HEVs [8]. Three additional putative ORFs of 280 - 600 nt that overlap with ORFs 1 or 2 were predicted for each rat HEV genome [8]. Recent studies have elucidated the characteristics of some of the less understood ORF encoded proteins using computational approaches to delineate their role in the pathogenesis of HEV [9, 11]. Therefore, the present study analyzed the ORF5 protein for its physiochemical properties, primary structure, secondary structure, tertiary structure and functional characteristics using bioinformatics tools.

## Materials and methods:

### Sequence retrieval:

The rat HEV ORF5 amino acid sequence (Accession number: GU345042) was retrieved from the NCBI (National Center for Biotechnology Information) GenBank database.

### Physicochemical properties analysis:

The amino acid sequences of the ORF5 protein in FASTA format was used as query in for the determination of physiochemical parameters. The various physical and chemical parameters of the retrieved sequences were computed using ProtParam (Expasy), a web-based server (ExPASy - ProtParam tool). The ProtParam tool employed various parameters such as, extinction coefficients (EC – protein-protein/protein-ligand interactions quantitative study) [12,14], half-life [15-19], instability index (II – protein stability) [20], aliphatic index (AI – relative volume occupied by protein's aliphatic side chains) [21], Grand Average of Hydropathicity (GRAVY - sum of all hydropathicity values divided by number of residues in a sequence) [22], theoretical pI and number of positive and negative residues.

### Structural analysis:

The primary structure of the ORF5 protein in terms of the percentage composition of amino acids was computed using the ProtParam (Expasy) tool and PSIPRED (PSIPRED Workbench (ucl.ac.uk). The self-optimized prediction method with alignment (SOPMA) software (npsa-prabi.ibcp.fr) and PSIPRED ((PSIPRED Workbench (ucl.ac.uk) were used to predict the secondary structure of the ORF5 protein. The prediction is based on a system of neural networks that combines the outputs from several original prediction methods (NORSnet, DISOPRED2, PROFbval and Ucon), with the evolutionary profiles and sequence features that correlate with the protein disorder such as predicted solvent accessibility and protein flexibility. Further, PSIPRED was also used to compute the secondary structure of the ORF5 protein. The tertiary structure of the target protein was modeled using the online program Phyre2 (http://www.sbg.bio.ic.ac.uk/phyre2). The generated ORF5 protein 3D model was validated using Ramachandran plot analysis (PROCHECK) (http://nihserver.mbi.ucla.edu/SAVES) for stereo-chemical property.

### Functional analysis:

Location of signal peptide cleavage in the ORF5 protein was predicted using Signal P-4.1 (SignalP - 5.0 - Services - DTU Health Tech). The N-linked sites for glycosylation were predicted using NetNGlyc 1.0 (http://www.cbs.dtu.dk/services/NetNGlyc/) server, provided by Centre for Biological Sequence Analysis, Technical University of Denmark (CBS DTU). The O-linked sites for glycosylation were predicted using NetOGlyc 4.0 (http://www.cbs.dtu.dk/services/NetOGlyc/) server, provided by Centre for Biological Sequence Analysis, Technical University of Denmark (CBS DTU). The phosphorylation sites were predicted using NetPhos3.1 (NetPhos - 3.1 - Services - DTU Health Tech) server, provided by Centre for Biological Sequence Analysis, Technical University of Denmark (CBS DTU). For phosphorylation studies, we performed both generic and kinase specific predictions. ANTHEPROT v.6.9.3 predicted phosphorylation and other modified sites in the ORF5 protein.

## Results and Discussion:

The rat HEV genome comprises six ORFs (ORF1, ORF2, ORF3, ORF4, ORF5 and ORF6) [8]. Although ORF5 is attributed to genomic component of HEV, its functional implication remains to be explored [8]. In the study presented here, we determined the functional and structural properties of the ORF5 encoded protein through assessing its physicochemical properties, primary structure, secondary structure, tertiary structure, post-translational modifications, motif prediction, sub-cellular localization and gene ontology analysis, using a set of different computational methods. The availability of the study sequence of the rat HEV consisting additional ORFs in GenBank facilitated us to explore the characteristics of the ORF5 protein. The physiochemical parameters are vital in deciphering the protein’s characteristics, thus were analyzed computationally. Some important physicochemical properties included aliphatic index, instability index and GRAVY value. The various physiochemical parameters of the ORF5 protein are summarized in Table 1(see PDF).

**Figure 1:**
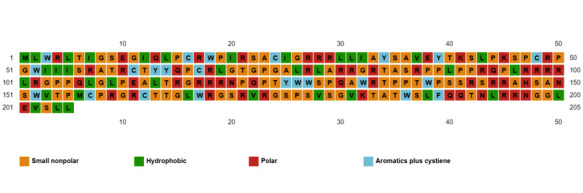
Representation of amino acid composition in ORF5 protein using PSIPRED.

Instability index governs the protein's characteristic [20]. A protein with instability index smaller than 40 is predicted as stable while a value above 40 is predicted as unstable [20]. Our higher instability index (>40) value indicated the unstable nature of the ORF5 protein [20]. The value of aliphatic index is another factor which governs the protein's thermal stability. A higher aliphatic index value suggests increased thermo-stability of the protein for a wide temperature range, as it is directly proportional to the thermal stability of the protein, i.e., proteins having higher aliphatic indices are comparatively more thermally stable in comparison to proteins having lesser aliphatic indices [21]. Thus, high aliphatic index value (84.33) suggested ORF5 to be a thermostable protein due to the presence of some aliphatic hydrophobic amino acids (Ile, Phe and Trp) [21]. Additionally, GRAVY is considered as an important factor for protein in determining its physiochemical properties. The value of GRAVY spread between - 0.310 and - 0.514 and lower values are suggested to have good interactions between water and protein [22]. Therefore, the ORF5 protein was found to be hydrophilic in nature (-0.141) (positive score indicated hydrophobicity). Thus, taken together it can be interpreted that the ORF5 protein was found to highly unstable, thermostable, hydrophilic and basic in nature. Proteins differ from one another in their structure, primarily in their sequence of amino acids. The linear sequence of the amino acid polypeptide chain refers to its primary structure. The amino acid composition of ORF5 protein is summarized in Table 2(see PDF) (Figure 1).

**Figure 2:**
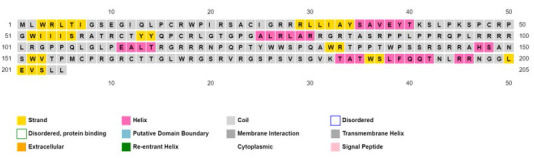
Secondary structure elements of ORF5 protein of rat HEV. The analysis was conducted using PSIPRED.

Arg was observed as the top amino acid that with the highest frequency. The top five amino acids that contributed to the polypeptide chain of ORF5 were included Arg, Pro, Leu, Ser and Gly (Figure 2). The default parameters (similarity threshold: 8; window width: 17) were considered by SOPMA for the secondary structure prediction with >70% prediction accuracy, utilizing 511 proteins (sub-database) and 15 aligned proteins. The predicted elements of secondary structure in the ORF5 proteins are mentioned in Table 3(see PDF). Thus, taken together, SOPMA predicted that the ORF5 protein consisted of all the three major elements of secondary structure, i.e., alpha helix (α), beta strand (β) and random coil. The predicted secondary structure elements by PSIPRED are shown in Figure 2.

The amino acids structural diversity plays a vital role in the formation of protein self-assembly. The three-dimensional spatial arrangement of amino acid residues in a protein is known as the tertiary structure. The secondary structure elements (helices and strands) are combined in different ways to form three-dimensional structures of a protein. To perform structure-based drug-designing, it is quite essential to build a reliable model. The generated 3D tertiary structure of the ORF5 protein (via Phyre2) was analyzed by visualization through homology modelling approach (Table 4 - see PDF).

**Figure 4:**
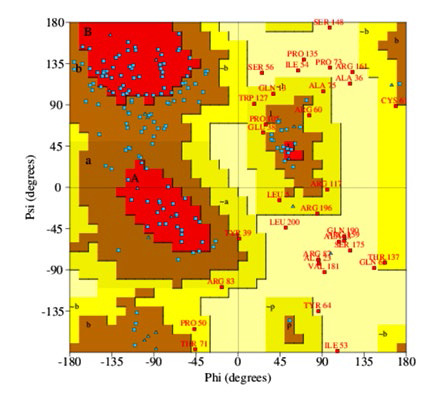
Ramachandran plot of the ORF5 protein of rat HEV showing the favoured regions. The analysis was conducted using PROCHECK.

Further, the obtained 3D model generated through Phyre2 was assessed using PDBsum and Ramachandran plot analysis (PROCHECK) (Figure 4). The overall protein's stereochemical quality, amino acids present in the allowed, disallowed region and the G-factor were evaluated by Ramachandran map (Table 5 - see PDF).

**Figure 5:**
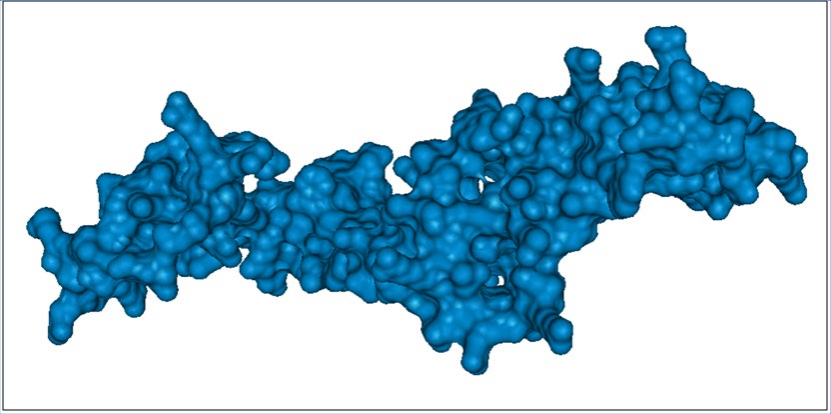
Surface representation of the modelled 3D structure of the ORF5 protein of rat HEV.

The model obtained from “RaptorX” was observed to be of a poor quality as it had a percentage favorable region of 37.9% and highly unusual value of G factor (-2.44) [23] (Table 4 - see PDF) (Figure 5B). The 3D structure modeled by Phyre2 also showed both α and β content with subsequently higher percentage of coils. Thus, our tertiary structural analysis was in agreement with the secondary structural analysis. Thus, it could be interpreted that the ORF5 protein domain is a mixed α/β structural-fold with predominance of coils. Moreover, the overall modelled ORF5 protein structure was irregular and revealed several clefts with two pores and a tunnel (Figure 5). Clefts are present on protein's surface which is important in the determination of protein interaction with the other molecules. The size of clefts is considered as primary factors in governing the interaction between the receptor proteins with the target molecules [24]. Tunnels are defined as access paths which connect the interior of the protein molecule to the surrounding environment and influence the process of the protein's reactivity [25]. Thus, the presence of clefts and tunnels also strengthens our analysis, suggesting the commitment of ORF5 protein towards interaction with the target molecules.

**Figure 6:**
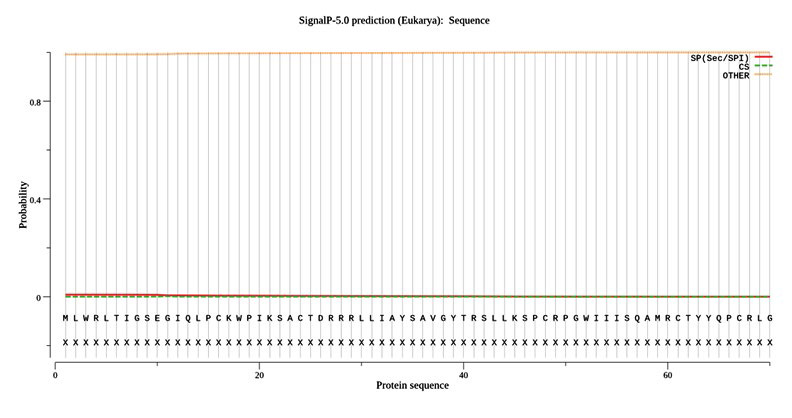
: SignalP-5.0prediction. Signal peptide likelihood was absent.

**Figure 7:**
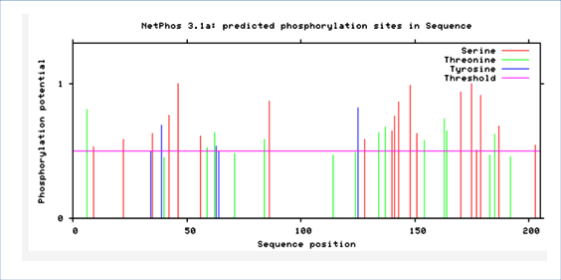
Predicted phosphorylation sites using NetPhos3.1

The potential cleavage site for signal peptide were found to be absent in the amino acid sequence (Figure 6). None of the N-linked sites for glycosylation was identified in the ORF5 protein. However, 17 O-linked possible sites for glycosylation were found using the NetOGlyc 4.0 server. Additionally, several phosphorylation sites including 19 Ser, 10 Thr and 3 Tyr residues were identified in the ORF5 protein using NetPhos3.1 server (Figure 7). Moreover, it was revealed that through ANTHEPROT that the ORF5 protein contained some modified sites such as, protein kinase C phosphorylation sites, casein kinase II phosphorylation sites and myristoylation sites, etc. The identified sites are mentioned in Table 5(see PDF).

Post-translational modifications (PTMs) are various different type of modifications such as, phosphorylation, glycosylation, ubiquitnation, acetylation, , etc. [26] and known to contribute to cellular signal transduction regulation, transcription and translation [27-29]. It is noteworthy to mention that our obtained ORF5 3D-model was identified with modified sites (glycosylation, phosphorylation, myristoylation and amidation). These are imperative prerequisite for proteins in order to carry out their various specific regulatory functions [30]. Presence of glycosylation sites have been shown to modulate the intracellular signaling machinery [29]. Additionally, protein phosphorylation constitutes an essential mechanism for the proper establishment of an infection cycle in several intracellular pathogens [30,31]. Furthermore, phosphorylation is required for protein folding, signal transduction, intracellular localization PPIs, transcription regulation, cell cycle progression, survival and apoptosis [30,32,33]. It has been documented in reports that attachment of a myristoyl group regulates cellular signaling pathways in several biological processes [28]. ORF5 protein could perform crucial regulatory functions by interacting with the other viral and host components. Data shows that the ORF5 protein plays critical role in the life cycle of rat HEV.

## Conclusion:

The Norway rat HEV ORF5 encoded protein is an essential component of its genome with unknown function. We document the physicochemical, structural and functional characteristics of the ORF5 encoded protein of Norway rat HEV using standard bioinformatics tools. The protein was highly unstable, thermostable, hydrophilic and basic in nature. The primary analysis revealed the higher abundance of the amino acids Arg, Pro, Leu, Ser and Gly. The secondary structural analysis revealed the presence of all three major components (helices, strands and coils). The 3D structure homology model showed the presence of mixed α/β structural fold with the predominance of coils. Further, the clefts, modified sites, such as phosphorylation, glycosylation, etc. signifies the importance of ORF5 protein in rat HEV pathogenesis. Knowledge on the structure of the ORF5 protein will provide insights into its functional role in the viral pathogenesis.

## Figures and Tables

**Figure 3:**
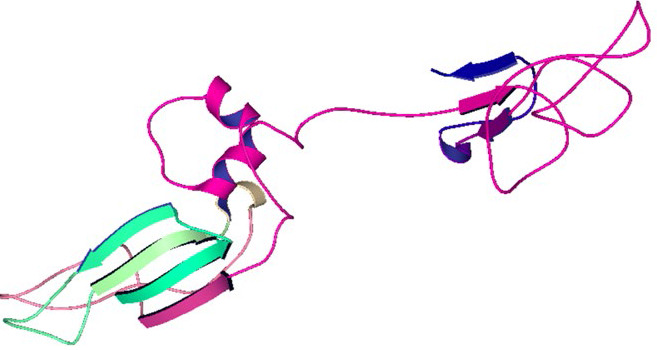
Tertiary structure of ORF5 protein of rat HEV. The analysis was conducted using Phyre2.
